# Chinese Preservice Teachers’ Professional Identity Links with Education Program Performance: The Roles of Task Value Belief and Learning Motivations

**DOI:** 10.3389/fpsyg.2016.00573

**Published:** 2016-04-26

**Authors:** Yan Zhang, Skyler T. Hawk, Xiaohui Zhang, Hongyu Zhao

**Affiliations:** ^1^School of Psychology, Beijing Normal UniversityBeijing, China; ^2^Department of Educational Psychology, The Chinese University of Hong KongHong Kong, China

**Keywords:** preservice teachers, teacher professional identity, task value belief, learning motivation, program performance

## Abstract

Professional identity is a key issue spanning the entirety of teachers’ career development. Despite the abundance of existing research examining professional identity, its link with occupation-related behavior at the primary career stage (i.e., GPA in preservice education) and the potential process that underlies this association is still not fully understood. This study explored the professional identity of Chinese preservice teachers, and its links with task value belief, intrinsic learning motivation, extrinsic learning motivation, and performance in the education program. Grade-point average (GPA) of courses (both subject and pedagogy courses) was examined as an indicator of performance, and questionnaires were used to measure the remaining variables. Data from 606 preservice teachers in the first 3 years of a teacher-training program indicated that: (1) variables in this research were all significantly correlated with each other, except the correlation between intrinsic learning motivation and program performance; (2) professional identity was positively linked to task value belief, intrinsic and extrinsic learning motivations, and program performance in a structural equation model (SEM); (3) task value belief was positively linked to intrinsic and extrinsic learning motivation; (4) higher extrinsic (but not intrinsic) learning motivation was associated with increased program performance; and (5) task value belief and extrinsic learning motivation were significant mediators in the model.

## Introduction

Professional identity has long been regarded as a key construct in teachers’ career development. It not only contributes to in-service teachers’ job outcome (e.g., job burnout, satisfaction) (Hong, 2010; Crocetti et al., 2014), but also predicts preservice teachers’ dedication to professional preparation. Examining responses from a cohort of pre-service teachers, Walkington (2005) argued that core beliefs and perceptions of teaching might affect the process of learning to teach and developing concepts of teaching. Observation of pre-service urban teachers (Merseth et al., 2008) also indicated the contribution of professional identity to engagement in teaching training. Beginning teachers that didn’t demonstrate formation of a strong professional identity during the training program experienced adjustment difficulties after they enter the occupation (Ewing and Smith, 2003; Ewing and Manuel, 2005). The studies suggested the significance of professional identity even in the pre-service stage of teachers’ career development, when they join an educational program. This study therefore focuses on preservice teachers’ professional identity, and the motivational processes that potentially underlie an association with educational preparation. We conducted this study on preservice teachers enrolled in the first 3 years of a 4-year teacher-training program in Mainland China.

In the educational phase, preservice teachers’ primary task is to prepare themselves to be skilled educators in the future. The quality of their preparation contributes to variation in their work outcomes. Compared to those without extensive preparation, teachers who follow educational training programs feel significantly more confident across most dimensions of teaching (Darling-Hammond et al., 2002). Educational preparation is also one of the most fundamental factors predicting teacher attrition (Chapman and Green, 1986). High teacher attrition rates in many countries are thought to have early roots in lower levels of the initial commitment fostered during the education and career preparation stage (Rots et al., 2007).

The key role of professional identity and the far-reaching impact of educational preparation suggest a need for explicit research on potential links between these variables. Specially, clarifying how professional identity links to teachers’ educational preparation might be helpful for improving program training quality. Importantly, professional identity might differ between the two main stages of training programs, i.e., the education stage and the initial practice stage. This study focuses on the first stage for two reasons. First, in the Chinese educational context, most preservice teachers must spend at least 3 years out of a 4-year program to finish their studies, including both subject major courses and teaching knowledge and skill courses. This phase of education contains only very limited, temporary practice activities. In contrast, the practice phase typically only lasts between 1 and 3 months. Thus the education stage is a crucial component of career preparation, and can to a large extent represent their performance in the program. Second, students’ performance in this initial educational phase is measured in terms of scores that are easily comparable, providing an objective method of evaluation. Therefore, in this study, we mainly focus on the initial education stage and explore the link between professional identity and program performance from a motivational perspective. We argue that motivational constructs including task value beliefs, and both intrinsic and extrinsic learning motivations, might mediate a link between professional identity and program performance. Task value beliefs reflect preservice teachers’ evaluations of whether what they are learning is important, useful, and interesting (Eccles et al., 2005). Learning motivation, whether provided through inherent interest or the promise of external reward, urges them to invest energy in study activities (Deci et al., 1999). We believe that these constructs might be involved in, and predict, preservice teachers’ engagement in educational training.

Researchers have utilized different conceptualizations of teachers’ professional identity across different studies and research disciplines (Beauchamp and Thomas, 2009), ranging from basic professional characteristics to teachers’ evaluations of their roles (for reviews, see Beijaard et al., 2004, and Beauchamp and Thomas, 2009). Importantly, the existing definitions often differ on aspects of uni-dimensionality vs. multi-dimensionality, continuity vs. discontinuity, and the individual vs. social nature of teaching (Akkerman and Meijer, 2011). Predominant perspectives have moved from conceptualizing professional identity as a uni-dimensional, stable attribute that is independent of social context, to a multi-faceted, dynamic process that develops continually based on the interaction with context over time (Beauchamp and Thomas, 2009; Hong, 2010). Considering these characteristics and functions, we define teachers’ professional identity as a dynamic, multi-dimensional psychological and behavioral orientation toward the teaching profession, based on related value judgments and emotional experiences. Specifically, we argue that professional identity consists of: (1) cognitions about the occupation’s characteristics, social functions, social status, and competence demands, which are basically external value cognitions and judgments that relate to the context; (2) emotional experiences and feelings related to professional work, reflecting internal evaluations of the profession; and (3) behavioral tendencies, in the form of willingness and commitment to engage in the teaching profession. Professional identity predicts teachers’ work attitudes and performance, including their teaching quality and innovation (Beijaard et al., 2004), job satisfaction (Moore and Hofman, 1988; Crocetti et al., 2014), turnover intention (Day et al., 2005; Hong, 2010), and pupils’ achievement in courses (Sammons et al., 2007), suggesting that it might hold crucial links to teachers’ career development.

Many studies examining models of teachers’ professional identity formation and development acknowledge that preservice teachers begin to establish an initial professional identity that is then continually modified after actually entering the occupation (e.g., Flores and Day, 2006; Luehmann, 2007; Beauchamp and Thomas, 2009). Its formation during the educational training period is also known as the “identity learning” process (Geijsel and Meijers, 2005), and is linked to factors including their beliefs and perceptions about the profession (Walkington, 2005), and their experiences as students and in pre-professional teaching (Hsieh, 2010). These factors contribute to the extended process of identity formation and development (Beijaard et al., 2000; Rodgers and Scott, 2008; Lamote and Engels, 2010). Beauchamp and Thomas (2009) argued that educational programs are the first important intervention point for helping teachers to develop their professional identity. Even though it further evolves in later stages, the initial identity formed during teacher education is foundational and crucial to future evolution. These findings lend additional support to the importance of explicitly studying links between professional identity and program performance in the educational stage, which has heretofore received scant research attention.

Although in-service teachers have been the focus of many prior studies (Lasky, 2005; Avalos and Aylwin, 2007; Hong, 2010; Pillen et al., 2013), other research also implied, sometimes indirectly, the importance of professional identity to preservice teachers’ growth and performance in educational training. Exploration and commitment, two guiding processes of identity status according to Marcia (1966), play key roles. Preservice teachers who reflected on their epistemological beliefs at the beginning of their education program, a form of exploration, showed more growth in sophisticated epistemological beliefs that could help them to improve understanding of teaching and learning (Brownlee et al., 2001). Additionally, commitment to the profession is predictive of students’ choice to actually enter into teaching (Rots et al., 2007; Hong, 2010), as well as of their positive feelings about future work (Porfeli et al., 2011). These results imply an association between preservice teachers’ higher professional identity and positive job-related behaviors. We therefore expect that *preservice teachers’ professional identity is positively linked to their performance in the teacher education stage (Hypothesis 1).*

Besides being regarded as a crucial factor in teachers’ educational training, professional identity – especially views about learning and teaching, and about oneself as a teacher – is also “the foundation for meaning-making” (Bullough, 1997, p. 21; cited from Lamote and Engels, 2010) and may have the function of motiving preservice teachers to engage in specific preparation activities. From this perspective, we argue that motivational constructs might also be involved in the process. We investigated task value belief, intrinsic learning motivation, and extrinsic learning motivation in this study.

Task value beliefs (i.e., task value or subjective task value, Atkinson, 1964; Feather, 1982; Eccles et al., 1993), originally derived from modern expectancy-value theory (for a review, see Wigfield and Eccles, 2000), consists of multiple components in prior studies, including attainment value (importance of doing well on the task), intrinsic interest (inherent pleasure from engaging in the task), utility value (value of a task for long-term and short-term goals), and perceived costs of engaging in the task (1983, as cited in Eccles et al., 2005). The link between professional identity and task value belief has attracted research attention, though prior studies remain limited. Task value belief is significantly correlated with professional identity in groups of teachers at different career stages. Dropout teachers, who have abandoned the profession, showed the lowest task value belief compared to both pre-service and in-service teachers (Hong, 2010). Hong’s (2010) study examined task value beliefs about teaching, however, which were thus interpreted as a component of professional identity. In other words, it did not examine behaviors that are part of the process of educational preparation. In the present research, we focus on the task of preparing to be a teacher, which is a specific behavior that can be considered more separable from one’s conception of actually being a teacher. We therefore take task value belief as a construct distinct from professional identity, and propose that *preservice teachers’ professional identity is positively connected with their task value beliefs about current learning (Hypothesis 2).*

Task value belief functions both to facilitate learning motivation and promote learning performance (Pintrich et al., 1993; for a review, see Wigfield and Cambria, 2010). On one hand, it is a force that motivates goal-directed behavior (Feather, 1982) and enables individuals to devote more energy to learning activities. Earlier research showed a connection between task value belief and college students’ intrinsic and extrinsic learning motivations (Pintrich et al., 1993; Eccles et al., 2005). Their valuing of learning tasks was a significant and positive predictor for their cognitive engagement and use of critical thinking in project-based courses (Stolk and Harari, 2014). On the other hand, decades of research has suggested that task value belief is a major predictor for academic achievement (Eccles et al., 2005). A longitudinal study indicated that task value belief was one of the best predictors of college students’ final course performance, even after controlling for prior performance (Zusho et al., 2003).

Based on the aforementioned research, we hypothesize that *preservice teacher’s task value belief predicts both their extrinsic learning motivation (Hypothesis 3) and intrinsic learning motivation (Hypothesis 4), and further predicts their program performance (Hypothesis 5)*.

Learning motivation is the psychological process that helps students to engage in goal-directed learning activities. Motivation can be distinguished on the basis of different reasons that give rise to an action (see Self-Determination Theory; Deci and Ryan, 1985), though they are typically correlated with each other. Intrinsic motivation refers to doing something because of inherent interest, and extrinsic motivation refers to doing something because of its separable outcome (Deci and Ryan, 1985; Ryan and Deci, 2000).

Two theories suggest a relationship between professional identity and learning motivation. First, identity-based motivation maintains a linkage of identity to identity-congruent action and cognitive procedures (for a review, see Oyserman, 2007, 2009). Former studies have shown that identity-based motivation is effective in influencing peoples’ health and consumer behavior (Oyserman et al., 2007; Oyserman, 2009). For preservice teachers, the desire to be a good teacher might supply them with an original motive to devote energy to learning activities that are congruent with the teaching profession. Second, possible-selves theory suggests that “the ideal selves that we would very much like to become” (Markus and Nurius, 1986, p. 954) influence the regulation of goal-directed and self-relevant behavior (Markus and Nurius, 1986; Erikson, 2007; Hamman et al., 2010), including academic initiative and school performance (Oyserman et al., 2002, 2006). Preservice teachers’ possible selves connecting to their professional identity might help them to get involved in learning activities for their future self (teacher) schema. However, this motivational effect is still not explored among preservice teachers, nor is the contribution of professional identity to their learning motivation fully understood (Hoyle and Sherrill, 2006; Oyserman, 2007). We therefore focus on this issue, and predict that that *preservice teachers’ professional identity is linked to both their extrinsic learning motivation (Hypothesis 6) and intrinsic learning motivation (Hypothesis 7)*.

Intrinsic and extrinsic learning motivations have shown links with school performance and persistence in study behaviors, and are therefore considered vital to students’ success (e.g., Vallerand and Blssonnette, 1992; Afzal et al., 2010; Akomolafe et al., 2013). Preservice teachers can be stimulated by both intrinsic and extrinsic learning motivations, although in different ways. Their passion for teaching and enthusiasm for the major are strong intrinsic motivations for high program performance, while their hope for a job in a good school and the salary and respect for being a teacher are examples of extrinsic motivations. Considering these differences, we examine the distinct associations that each type of motivation holds with professional identity, task value belief, and program performance. We expect that both *extrinsic learning motivation (Hypothesis 8), and intrinsic learning motivation (Hypothesis 9), are linked to preservice teachers’ program performance.*

**FIGURE 1:**
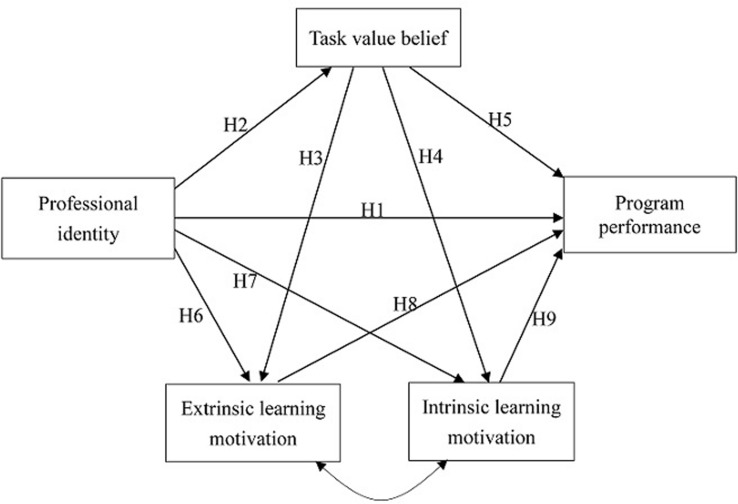
**A suggested model on student teachers’ professional identity, task value belief, extrinsic learning motivation, intrinsic learning motivation, and program performance**.

In a suggested model examining teacher attrition (Chapman, 1984), teacher professional identity is a key factor that influences program performance and further contributes to the decision to leave teaching. However, empirical evidence for the psychological processes that might account for this link is still needed. Based on the prior research reviewed here, we assume that motivational constructs (task value belief, intrinsic learning motivation, and extrinsic learning motivation) might play key roles in this regard. To date, however, prior studies have offered only piecemeal evidence for such processes, without providing an integrated account of these links. The present study aims to investigate an overall model examining the direct and indirect relationships between professional identity, motivational constructs, and program performance among preservice teachers, a heretofore under-examined population. Based on *Hypotheses 1*–*9*, we propose a model establishing the links between these variables (see **Figure 1**). Exploring these relations can offer information that might be crucial to improving preservice teachers’ educational performance, and provide implications for teacher training.

## Materials and Methods

### Participants

The present study was carried out with 625 preservice teachers, and 606 were left as valid data after excluding those providing irregular answers or missing too many items. Of the participants, 28.7% were males (*n* = 174) and 67.98% were female (*n* = 412), with 20 not reporting their gender. All of them had been trained as preservice teachers for less than 3 years, with first year students making up 37.95% (*n* = 230), sophomores 36.80% (*n* = 223), juniors 25.08% (*n* = 152), and one who did not report a program year. The sample spanned eight subject domains (physics, chemistry, foreign language, liberal arts, psychology, education technology, special education, and preschool education). As preservice teachers, they were trained on both major knowledge and teaching skills. The study was approved by the Ethics Committee of the School of Psychology, Beijing Normal University, and all the work was carried out within the guidelines set by the committee.

### Measures and Materials

#### Teacher Professional Identity

A previously validated scale used in prior research with mainland Chinese samples measured preservice teachers’ professional identity (Zhang et al., 2011; Zhao et al., 2012). It consists of 15 items, for which students responded on a 4-point Likert self-report scale (1 = *strongly disagree*, 2 = *disagree*, 3 = *agree*, and 4 = *strongly agree*). Three dimensions were contained: intrinsic value identity, mainly related to individuals’ subjective feelings toward the teaching profession (seven items, e.g., *I like teaching*), extrinsic value identity, mainly focused on cognitions about external factors of the teaching profession (three items, e.g., “*I think the work environment and condition for teacher is great*”), and volitional behavior identity, indicating their behavioral engagement and willingness to join the profession (five items, e.g., “*I often actively participate in trainings and lectures for teacher and teaching for promotion*”). The whole scale showed acceptable reliability, Cronbach α = 0.78, as did each dimension (Cronbach α = 0.88, 0.78, and 0.75, respectively). The correlations between each item and the total score of the scale are presented in **Table 1**.

**Table 1:** Items of teacher professional identity scale and their correlation with the total score.

	Items	Correlation with the total score	Dimensions
1	I think teacher is a rewarding profession.	0.63^∗∗^	a
2	I think the development space for teacher profession is big.	0.67^∗∗^	a
3	I like teaching.	0.77^∗∗^	a
4	I admire the way the teacher live and work.	0.75^∗∗^	a
5	I think it’s happy for a teacher to communicate with students.	0.66^∗∗^	a
6	I think teacher’s work is very interesting.	0.73^∗∗^	a
7	I think being a teacher is fulfilling/brings sense of achievement.	0.72^∗∗^	a
8	I frequently pay attention to the information about teacher profession, including policies on welfare, medical service, and opportunity for advanced courses taking and so on.	0.49^∗∗^	c
9	I often actively participate in trainings and lectures for teacher and teaching for promotion.	0.58^∗∗^	c
10	I often read books that related to teacher and teaching.	0.56^∗∗^	c
11	Even if there is no policy constraint (e.g., future job assigning), I will still choose to be a teacher.	0.69^∗∗^	c
12	If I choose to be a teacher in the future, I will choose to work as a teacher all my life.	0.65^∗∗^	c
13	I think the work environment and condition for teacher is great.	0.56^∗∗^	b
14	I think teachers’ social status is high.	0.58^∗∗^	b
15	Teacher is a highly respected occupation.	0.55^∗∗^	b

Dimensions: a, intrinsic value identity; b, extrinsic value identity; c, volitional behavior identity. **p ≤ 0.01.

#### Task Value Belief

A scale created by Pintrich et al. (1993) measured task value belief with six items scored on a 4-point Likert scale (1 = *strongly disagree*, 2 = *disagree*, 3 = *agree*, and 4 = *strongly agree*). It has previously showed high validity and reliability in measuring student’s task value belief in learning (Pintrich et al., 1993). In this study, we slightly changed the items by extending the target to learning in the program, but not limited to only one specific course (e.g., in the item “*I think learning in this program is useful for me,” “learning in this program”* replaced *“the course material in this class”*) The Cronbach α for the scale was 0.87, demonstrating good internal consistency. Factor scores and reliability were highly similar to those reported in prior research (Pintrich et al., 1993), with the loadings of the items varying from 0.56 to 0.85.

#### Learning Motivation

We measured learning motivation using the intrinsic goal orientation and the extrinsic goal orientation scales of the Motivated Strategies for Learning Questionnaire (MSLQ, Pintrich et al., 1993), which have been used as motivation measures in former research (e.g., Stolk and Harari, 2014). The scales were previously used in conjunction with the present task value beliefs scale (Pintrich et al., 1993). Each of the two scales contained four items, measured on a 4-point Likert scale (1 = *strongly disagree*, 2 = *disagree*, 3 = *agree*, and 4 = *strongly agree*). We also extended the items to represent more basic learning activities in the program as opposed to one specific class (e.g., in an item for intrinsic measure, “*In a class like this, I prefer course material that arouses my curiosity, even if it is difficult to learn*,” the phrase “*in a class like this*” was deleted in our study; In an item for extrinsic measure, “*I want to do well in this class because it is important to show my ability to my family, friends, employer, or others*” for extrinsic measure, the phrase “*in this class*” was replaced by “*in my studies*”). Reliabilities of these subscales were sufficient, Cronbach α = 0.72 and 0.76, respectively. The scores of the two scales were used separately as indicators for intrinsic learning motivation and extrinsic learning motivation.

#### Program Performance in the Education Stage

In this study, we used the students’ grade-point average (GPA) to represent their program performance in the education stage. We employed it both due to realistic and theoretical considerations. Realistically, in Chinese teacher education programs, all the students are dedicated to completing university courses for at least three out of 4 years of the program. The courses included both those related to their majors and those related to teaching knowledge and skills. Some of the teaching skill courses also included short-term activities of teaching in schools. All the courses were measured in terms of final grades. Therefore, the GPA can reasonably be employed as an indicator of how preservice teachers performed and learned in this stage. Theoretically, GPA provides an objective measure for how preservice teachers prepared in their knowledge and teaching skills across subjects. In contrast, the practicums they would join in the fourth year would only last between 1 and 3 months, and are not assessed by grades but instead on five ranks (from excellent to unqualified) by different target schools in different districts where they finish the practicum. Although we do not deny the reference value of the evaluation they receive from the practicum, we believe that under the Chinese teacher training scheme, 3 years of study should robustly reflect how preservice teachers are trained in the program. Thus, we regard GPA to be a useful indicator. As the preservice teachers came from different departments, we calculated z-standardized scores within each department of each program year, in order to avoid possible bias before joining the data for further analysis.

### Statistical Analyses

We included the three dimensions to construct the latent variable of teacher professional identity in a structural equation model (SEM; Mplus v.7; Muthén and Muthén, 1998), and used it to predict program performance (Model 1). In stages, we added task value belief, intrinsic learning motivation, and extrinsic learning motivation as independent (Model 2) and correlated (Model 3) mediators. Bootstrapping (5000 resamples) was used to check the strengths and significances of the indirect paths. Acceptable fit for SEM was set at CFI ≥ 0.90, and RMSEA and SRMR ≤ 0.10 (Kline, 2010).

## Results

Means, standard deviations, and correlations between the three dimensions of preservice teacher’s professional identity, task value belief, intrinsic learning motivation, extrinsic learning motivation, and program performance can be seen in **Table 2**^1^. The three dimensions of preservice teacher’s professional identity were positively correlated with each other. Other study variables were also significantly associated with each other, except for the correlation between intrinsic learning motivation and program performance.

**Table 2:** Means, standard deviations, and within-subject correlations between the study variables.

		*M*	*SD*	1	2	3	4	5	6
1	Intrinsic value identity	3.04	0.55						
2	Extrinsic value identity	2.76	0.61	0.50^∗∗^					
3	Volitional behavior identity	2.56	0.56	0.66^∗∗^	0.39^∗∗^				
4	Task value belief	3.09	0.53	0.40^∗∗^	0.27^∗∗^	0.24^∗∗^			
5	Intrinsic learning motivation	3.18	0.47	0.36^∗∗^	0.25^∗∗^	0.22^∗∗^	0.51^∗∗^		
6	Extrinsic learning motivation	2.80	0.53	0.28^∗∗^	0.19^∗∗^	0.30^∗∗^	0.41^∗∗^	0.31^∗∗^	
7	Program performance (z score)						0.10^∗^	0.04	0.19^∗∗^

*N = 606. ^∗^p ≤ 0.05, ^∗∗^p ≤ 0.01.*

To find the best fitting model, we tested our model step by step, from the basic main effect model (Model 1) to the hypothesized multiple mediation model (Model 3).

### Model 1

**FIGURE 2:**
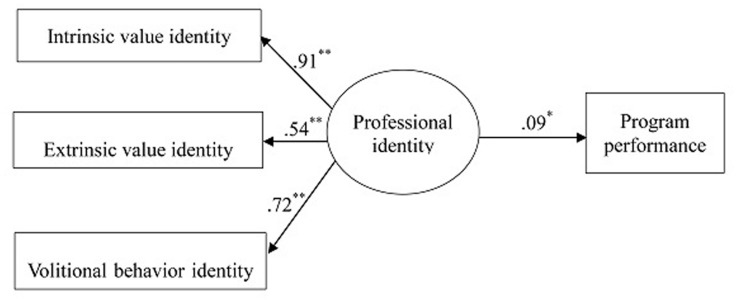
**Basic Model (Model 1).** Model fit: χ^2^(2) = 2.07, *p* = 0.35, CFI = 1.00, RMSEA = 0.008, 90% CI = 0.00 -0.08, SRMR = 0.01. ^∗^*p* ≤ 0.05, ^∗∗^*p* ≤ 0.01.

A basic model using teacher professional identity as a latent variable tested the link between professional identity and program performance (Model 1, see **Figure 2**^2^). The results yielded an acceptable model fit: χ^2^(2) = 2.07, *p* = 0.35, CFI = 1.00, RMSEA = 0.008, 90% CI = 0.00 -0.08, SRMR = 0.01. It showed a significant link between teacher professional identity and program performance, *B* = 0.15, *SE* = 0.07, *p* = 0.03, and all the three dimensions of teacher professional identity showed acceptable loadings on the latent variable (loadings were 0.91, 0.54, and 0.72).

### Model 2

**FIGURE 3:**
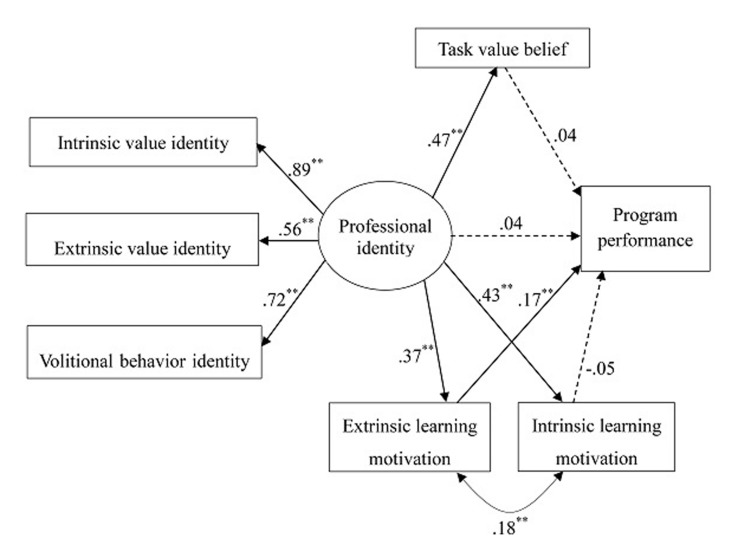
**Independent Model (Model 2).** Model fit: χ^2^(10) = 172.71, *p* < 0.001, CFI = 0.84, RMSEA = 0.16, 90% CI = 0.14 -0.19, SRMR = 0.07. ^∗∗^*p* ≤ 0.01.

We further tested Model 2 (see **Figure 3**), adding task value belief, intrinsic learning motivation, and extrinsic learning motivation into the model. This new model showed an unacceptable model fit, χ^2^(10) = 172.71, *p* < 0.001, CFI = 0.84, RMSEA = 0.16, 90% CI = 0.14 -0.19, SRMR = 0.07. It also showed no significant link between professional identity and program performance (*B* = 0.07, *SE* = 0.09, *p* = 0.47). Teacher professional identity showed significant links with task value belief (*B* = 0.51, *SE* = 0.05, *p* < 0.001), intrinsic learning motivation (*B* = 0.41, *SE* = 0.04, *p* < 0.001), and extrinsic learning motivation (*B* = 0.43, *SE* = 0.40, *p* < 0.001). In addition, program performance showed significant link with extrinsic learning motivation (*B* = 0.26, *SE* = 0.07, *p* < 0.001). Tests of indirect effects indicated that extrinsic learning motivation significantly mediated the link between teacher professional identity and performance (β = 0.10, *p* = 0.001). Modification indices provided by Mplus suggested that the model fit could be further improved by including links from task value belief to intrinsic learning motivation and to extrinsic learning motivation, which were in line with our theoretical expectations. We employed these additional links in Model 3.

### Model 3^3^

**FIGURE 4:**
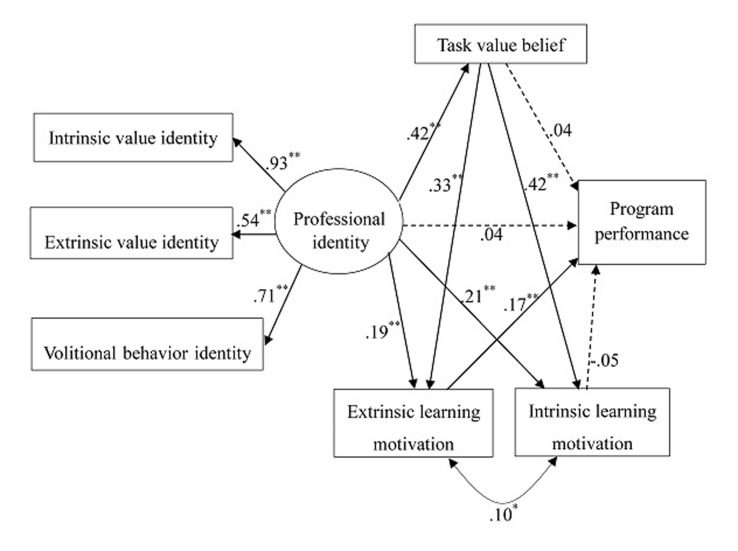
**Full Model (Model 3).** Model fit: χ^2^(8) = 28.10, *p* = 0.0005; CFI = 0.98, RMSEA = 0.06, 90% CI = 0.04 -0.09, SRMR = 0.02. ^∗^*p* ≤ 0.05, ^∗∗^*p* ≤ 0.01.

In the third model, we added the links from task value belief to intrinsic learning motivation and to extrinsic learning motivation (see **Figure 4**). The model showed an acceptable fit, χ^2^(8) = 28.10, *p* < 0.001; CFI = 0.98, RMSEA = 0.06, 90% CI = 0.04 -0.09, SRMR = 0.02. All the three dimensions of teacher professional identity showed acceptable loadings on the latent variable (loadings were 0.93, 0.54, and 0.71). As expected, teacher professional identity was significantly correlated with task value belief (*B* = 0.44, *SE* = 0.05, *p* < 0.001), intrinsic learning motivation (*B* = 0.19, *SE* = 0.05, *p* < 0.001), and extrinsic learning motivation (*B* = 0.19, *SE* = 0.06, *p* < 0.001). Task value belief showed links with intrinsic learning motivation (*B* = 0.37, *SE* = 0.04, *p* < 0.001) and extrinsic learning motivation (*B* = 0.33, *SE* = 0.05, *p* < 0.001). In addition, intrinsic and extrinsic learning motivations were modestly but significantly correlated (*B* = 0.02, *SE* = 0.009, *p* = 0.04). As in Model 2, program performance showed a link only with extrinsic learning motivation (*B* = 0.27, *SE* = 0.06, *p* < 0.001). We used bootstrapping with 5000 resamples to test the mediating effects of extrinsic learning motivation and task value belief in the model. Results demonstrated that: (1) extrinsic learning motivation mediated a link between teacher professional identity and program performance (β = 0.03, *p* = 0.007); (2) task value belief mediated a link between teacher professional identity and intrinsic learning motivation (β = 0.18, *p* < 0.001); (3) task value belief mediated a link between teacher professional identity and extrinsic learning motivation (β = 0.14, *p* < 0.001); (4) extrinsic learning motivation mediated a link between task value belief and program performance (β = 0.06, *p* < 0.001).

## Discussion

In this study, we focused on preservice teachers’ professional identity and its link to program performance. We also included three motivational constructs, namely task value belief, intrinsic learning motivation, and extrinsic learning motivation, and explored their connections with professional identity and program performance. Our results indicated that: (1) preservice teachers’ professional identity was significantly associated with their program performance (in support of H1) and task value belief (in support of H2); (2) task value belief was linked to extrinsic learning motivation (in support of H3) and intrinsic learning motivation (in support of H4); (3) professional identity was also significantly associated with extrinsic learning motivation (in support of H6) and intrinsic learning motivation (in support of H7); (4) extrinsic learning motivation was connected with program performance (in support of H8). Two pathways were not supported in our model, however, namely the link between task value belief and performance (H5), and that between intrinsic learning motivation and performance (H9).

We also found four significant mediating effects: (1) extrinsic learning motivation mediated the connection between professional identity and program performance; (2) task value belief mediated the link between professional identity and extrinsic learning motivation, and (3) task value belief mediated the association between professional identity and intrinsic learning motivation; (4) extrinsic learning motivation mediated a link between task value belief and program performance.

### Relations between Teacher Professional Identity, Motivational Constructs, and Preservice Teachers’ Program Performance

As professional identity is a dynamic process that changes along with the context and teachers’ own growth (Geijsel and Meijers, 2005; Korthagen and Vasalos, 2005), it is essential to clarify its connection to performance at each stage of career development. This study, from a more integral perspective, revealed its important links to preservice teachers’ performance in education training programs. The conclusion is in line with Chapman’s (1984) model, which claimed that educational preparation and professional identity (initial commitment to teaching) interact in a cyclical fashion to contribute to the final teaching career choice. In light of this, preservice teachers’ professional identities should be considered even at the beginning, when they join the training program. Developing a strong identity provides them with an impetus to do a better job in educational preparation for their future occupation. On the basis of this study, we suggest that a weaker professional identity might be one of the reasons for preservice teachers’ lack of interest in profession-related work and lower focus on developing related skills. In fact, support for an identity-academic performance link has also been found with regard to other social identities, such as ethnic group and endorsement of gender stereotypes (Steele, 1997; Shih et al., 1999). Our findings extended evidence for an identity-academic performance effect to professional identity. The results supported the proposition that identity does matter for education program outcomes (Steele, 1997; Wheeler and Petty, 2001), in this case with regard to preservice teachers.

In contrast to earlier research, the present study included important motivational constructs that might account for the link between professional identity and preservice teachers’ program performance. The mediating roles of task value belief in the link between professional identity and learning motivation (both intrinsic and extrinsic), and of extrinsic learning motivation in the links both between identity and performance, and between task value belief and performance, suggested that professional identity might function to promote educational preparation in an indirect way through these motivational constructs. The results were in line with Oyserman’s (2007, 2009) identity-based motivation theory, which suggested that identity functions stimulate relevant procedure or action readiness. From this perspective, professional identity shifts preservice teachers into a profession-facilitating psychological state, and motivates them to engage related behaviors, such as professional preparation. This study further supported the notion that identity serves important functions not only for in-service teachers’ job outcomes (Crocetti et al., 2014), but also for preservice teachers’ mental processes and activities related to career preparation. It implies an explanation or alternative pathway for the effects of identity upon profession-related behaviors.

The link between task value belief and program performance was not supported in this study, however, although they were connected through extrinsic learning motivation. This finding is different from prior research using other samples (Pintrich et al., 1993; Eccles et al., 1998, 2005; Zusho et al., 2003). In former studies, researchers asked students to indicate the degree to which they valued a certain domain or a specific class (e.g., math, reading, or sports) (e.g., Pintrich, 1999; Eccles et al., 2005; Stolk and Harari, 2014), meaning they could give the value score on a specific domain based on students’ perceptions, which might increase the predictive value of performance in a specific subject. In contrast, our study took a more general perspective on participants’ task value belief. We also took the average score of courses as an indicator of program performance, instead of examining the score of a specific course. This might contribute to the non-significant direct link between task value belief and performance. Nevertheless, task value belief showed a link with performance through extrinsic learning motivation, implying that it may arouse preservice teachers’ desire for extrinsic reward, and this further contributes to their program performance.

We found that extrinsic learning motivation was directly linked to preservice teachers’ program performance, but intrinsic learning motivation was not. Reviewing former studies, diverse relations between learning motivations and performance have been reported. Some have indicated that both motivation dimensions significantly predict students’ GPA or course grades, while others implicate only one dimension (e.g., Pintrich et al., 1993; Fortier et al., 1995; Deci et al., 1999; Stolk and Harari, 2014), and still others have found no effects of either dimension (e.g., Pintrich, 2000; Bartels et al., 2011). When comparing findings across diverse samples and studies, Pintrich (2000) found that extrinsic learning motivation showed different effects, with some studies showing positive links with performance across stages, and others is showing negative links. This variation might due to the characteristics of particular samples and/or their learning patterns. Such factors might also have contributed to the relations between learning motivation and program performance in the present study. In college, preservice teachers have much more choice and control over their learning activities, including content, time, effort, and even class attendance. Under such conditions, their eagerness for external reward such as a higher GPA might promote class attendance, engagement with course-related work, and, ultimately, better performance. While interest in a major or the program is not necessary to facilitate course attendance or grade improvement, considering that universities provide students with many resources that are extra-curricular or not related to coursework. Apart from college students’ learning and study habits, two contextual factors could also contribute to the results. First, the Chinese education system places a strong emphasis on competition and performance rankings, which might reinforce students’ external motivation to study. Even in college, preservice teachers might still adhere to this motivational view. Second, the participants in this study were all covered by the free education policy for normal university students in China that has been in place since 2007. The policy states that all individuals covered by the policy receive benefits including free tuition during the training program, living allowance for 4 years of college education, and post-graduate placement in the national higher education system, albeit with some restrictions (e.g., service at rural districts for years). Thus, some of the participants might strongly value this external reward, and could possibly be more orientated toward external goals in the program. In addition, theoretically, we suggest that in Chinese culture, preservice teachers’ professional identity might be more orientated toward external reward in the absence of practical experience. Their deeper emotional connection to the profession might more likely develop after they enter the workforce. Under such conditions, profession-related motivations might also be more externally orientated in this stage, and would evolve as the professional identity changes with additional work experience. More research comparing different stages is needed to support these notions. Nevertheless, the different associations between intrinsic and extrinsic learning motivation with professional identity and education performance indicates the importance of extrinsic learning motivation to the preservice teachers in this study.

### Value and Contribution of the Current Study

In contrast to studies that have narrowly focused on professional identity itself, including its formation (e.g., Goodson and Cole, 1994; Cattley, 2007; Dang, 2013) and related identification processes (e.g., Beijaard, 1995), this study expanded the scope to link professional identity to program performance in career preparation programs. Additionally, our motivational perspective has important implications for the psychological processes that might underlie this association. Generally, this study makes three main contributions. Theoretically, it provides an integral perspective to understand how preservice teachers’ professional identity might predict the quality of their educational performance, and how the motivational constructs play a role in the process. Our findings complement past theories, such as Chapman’s (1984) model. This helps us to understand why professional identity is important even at the early stage (i.e., training program) of teachers’ career development. Methodologically, we enriched the existing literature of the domain using a quantitative approach with a large sample size, and contributed support for the importance of preservice teachers’ professional identity in addition to evidence from interviews or observations (e.g., Cattley, 2007; Hong, 2010; Hsieh, 2010). Practically, it provides evidence-based suggestions for improving preservice teachers’ preparation for their future career, in terms of strengthening their professional identity and the related mediators. Policies related to preservice teacher selection and educational curricula should include considerations of preservice teachers’ professional identity and the factors that might draw them to this occupation. Importantly, however, it is highly like that cross-cultural variation exists in these associations, in terms of both participants’ characteristics and the educational program designs. Therefore, more cross-cultural research is warranted.

### Limitations and Future Directions

Naturally, there are still limitations in this study that can be improved in future research. First, the correlational nature of our data prevents the identification of causal effects between the variables. While it would be practically challenging, and perhaps ethically questionable, to experimentally manipulate some of the variables in our model, preservice teachers who receive intervention programs aimed at strengthening professional identity could be compared to those who receive no such intervention. Longitudinal studies following preservice teachers over time could also give insight into the developmental order of these links, although such research also cannot speak to the issue of causation. Second, the study was carried out in mainland of China, and the sample was covered by free education policy for normal university students. Thus, this model should be further examined using samples from other countries, including those that might differ in terms of preservice educational structure or extrinsic benefits. Third, we didn’t include students in the fourth year that had undergone their teaching practicum. As actual teaching practice might exert influences on professional identity, it would be valuable to also include students in 4th year. Finally, other psychological process variables, such as self-efficacy, learning styles, and emotional experiences could also be considered in future research.

## Author Contributions

YZ: substantial contributions to the data analysis, interpretation of data for the work, and draft the work for important intellectual content; SH: Substantial contributions to the data analysis, interpretation of data for the work, and draft and revise the work for important intellectual content; XZ: substantial contributions to the conception and design of the work, organization for study implementation, data collection, data analysis, interpretation for the results, and also the implications for practical work. HZ: substantial contributions to the conception or design of the work.

## Conflict of Interest Statement

The authors declare that the research was conducted in the absence of any commercial or financial relationships that could be construed as a potential conflict of interest.

## Footnotes

1 Since a latent variable of teacher professional identity was used in the SEM model, we didn’t include the dimensions’ correlations with other variables in this model.

2 All estimates in Figures are standardized.

3 Using a multi-group (MG) analysis, we additionally explored whether the paths in the final model differed according to the participants’ number of years in their program (Year 1, Year 2, or Year 3). We began with a model in which all the pathways were free between the groups (the variance of one factor loading for each group was constrained to 1.0 to allow for an identified model) (MG-M_0_) to get a comparable model for further testing [χ^2^(28) = 78.937, *p* < 0.001; CFI = 0.950, RMSEA = 0.095, 90% CI = 0.071 -0.120, SRMR = 0.040]. In order to examine the equivalence of the loadings on our latent professional identity variable, we then tested a model in which these three factor loadings were constrained between groups while all other pathways were allowed to vary (MG-M_1_) [χ^2^(32) = 83.787, *p* < 0.001; CFI = 0.950, RMSEA = 0.090, 90% CI = 0.066 -0.113, SRMR = 0.048). Imposing these constraints did not worsen model fit in comparison to MG-M_0_ (^Δ^χ^2^ = 4.85, ^Δ^df = 4, *p* = 0.303). In a final test (MG-M_2_), we additionally constrained all remaining regression paths. We chose this approach because we had no theoretical rationale for prioritizing the constraining of some paths over others in an incremental manner. This model [χ^2^(52) = 109.233, *p* < 0.001; CFI = 0.944, RMSEA = 0.074, 90% CI = 0.054 -0.093, SRMR = 0.088] did not show a significantly worse fit in comparison to MG-M_1_ [^Δ^χ^2^ = 25.446, ^Δ^df = 20, *p* = 0.185]. We therefore concluded that it was more parsimonious to examine the model including all participants, without distinguishing separate groups.
